# Accessing genetically defined cell types in the superior colliculus with transgenic mouse lines

**DOI:** 10.1016/j.isci.2025.112194

**Published:** 2025-03-11

**Authors:** Chen Chen, Yuanming Liu, Jianhua Cang

**Affiliations:** 1 Department of Psychology, University of Virginia, Charlottesville, VA 22904, USA; 2 Department of Biology, University of Virginia, Charlottesville, VA 22904, USA

**Keywords:** Systems neuroscience, Neuroanatomy, Transcriptomics, Model organism

## Abstract

Recent studies have revealed diverse neuron types in the superior colliculus (SC), a midbrain structure critical for sensorimotor transformation. Here, as an important step toward studying the function of these subtypes, we characterize 10 transgenic mouse lines based on a recently published molecular atlas of the superficial SC. We show that Cre or fluorescence expression in some lines corresponds specifically to certain transcriptomic neuron types. These include two GENSAT lines that have been used to target morphological cell types in the SC and three knockin lines. In contrast, such a correspondence is not seen in other tested mice. Importantly, the expression pattern of marker genes in all these lines is highly consistent with the molecular atlas. Together, our studies support a correlation between morphological and transcriptomic neuron types, identify useful lines for targeting SC neuron types genetically, and demonstrate the validity of the single-cell transcriptomics data.

## Highlights

• We characterized 10 transgenic mouse lines in the SC based on transcriptomic atlas
• Mouse lines marking WFVs or NFVs label distinct transcriptomic neuron types
• We identified useful mouse lines for targeting SC neuron types genetically
• Other lines do not specifically label SC transcriptomic neuron types as predicted

## Graphical abstract

**Figure:**
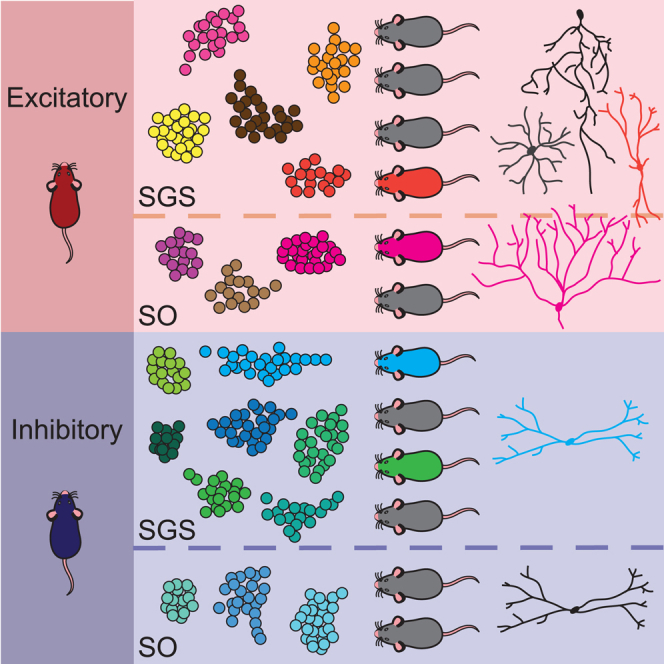

## Introduction

The nervous system is composed of a vast array of neuron types.^1^^,^^2^ Identifying and studying such neuron types is crucial for understanding brain function, development, and evolution.^3^^,^^4^ The recent development and application of single-cell genomics have provided rich information of gene expression with unprecedented resolution and detail in understanding neuronal heterogeneity.^5^ These studies have generated molecular atlases of diverse neuron types in many brain structures.^6^^,^^7^^,^^8^^,^^9^^,^^10^^,^^11^ By revealing type-specific molecular markers, they have also identified genetic access points for studying specific neuronal populations, which in turn facilitates the use of transgenic mice to study the function of individual molecular cell types.^12^^,^^13^^,^^14^ By leveraging type-specific gene expression and strategically selecting transgenic mice, this approach is a powerful tool for investigating the complexities of the nervous system at genetic, molecular, cellular, and behavioral levels.

In this study, we evaluate several transgenic mouse lines that potentially label specific neuron types of the superior colliculus (SC). The SC is a conserved midbrain structure in all vertebrates.^15^^,^^16^ It integrates sensory information, coordinates motor responses, and plays a pivotal role in guiding behavior and attention in response to the environment.^17^^,^^18^^,^^19^^,^^20^^,^^21^ Supporting its complex functions, the SC is divided into horizontal layers, and each of them serves different functions.^22^ The superficial layers of the SC (sSC) mainly process visual information, which receive direct inputs from the retina and visual cortex.^23^^,^^24^^,^^25^^,^^26^^,^^27^^,^^28^ Previous studies have classified sSC neurons into different types based on their morphology, such as wide-field vertical (WFV), narrow-field vertical (NFV), stellate, and horizontal cells.^29^^,^^30^^,^^31^^,^^32^ Studies in mice have already benefited from available transgenic mouse lines that serendipitously target certain morphological types. For example, Ntsr1-GN209-Cre and Grp-KH288-Cre lines from GENSAT^33^^,^^34^^,^^35^ were found to label WFV and NFV cells, respectively.^29^ These mouse lines were subsequently used to reveal how these cell types contribute to visual processing and visually guided behaviors.^29^^,^^30^^,^^36^^,^^37^ More diverse neuron types have now been revealed in the SC by single-cell transcriptomics.^12^^,^^14^^,^^38^^,^^39^^,^^40^ For example, our lab performed single-nucleus RNA sequencing (snRNA-seq) of sSC neurons and classified them into 28 neuronal clusters, which included 10 excitatory and 18 inhibitory ones.^38^ As in other systems, the information of transcriptomic neuron types in the sSC enables studies to answer fundamental questions regarding its organization, function, and development.

Here, as an important step in such studies, we have characterized a number of transgenic mouse lines to determine if they could target specific neuron types in the sSC. We first evaluated the molecular identity of Cre-expressing SC neurons in the Ntsr1-GN209-Cre and Grp-KH288-Cre mice using snRNA-seq or RNA fluorescence *in situ* hybridization (FISH). Our results demonstrate that the Cre-expressing sSC neurons in both lines indeed fell into distinct transcriptomic clusters with expression of specific marker genes. Next, we screened several other transgenic mouse lines, mostly knockin Cre mice, that were targeted to loci of marker genes expected to correspond to specific sSC neuron types according to the sSC snRNA-seq atlas. We found that the Cre expression in several of these lines specifically targets sSC neurons that express corresponding marker genes, whereas a lack of correspondence was seen in others. Importantly, in all the tested mouse lines, we verified that the expression pattern of each marker gene, including their layer distribution and excitatory/inhibitory nature, is highly consistent with the snRNA-seq atlas. Together, our results demonstrate the validity of the single-cell transcriptomics data, support a correlation between morphological and transcriptomic neuron types in the sSC, and highlight the importance of careful verification of transgenic mice. Our comprehensive analysis of gene markers for different molecular cell types and their corresponding transgenic mice can be a useful resource to guide future studies of sSC functions.

## Results

We previously performed snRNA-seq in the mouse sSC and identified 28 transcriptomic clusters of neurons.^38^ In this atlas, neurons in Clusters n02 to n11 (10 clusters) express vesicular glutamate transporter 2 (vGluT2) and are excitatory, whereas neurons in Clusters n01 and n12 to n28 (18 clusters) express vesicular GABA transporter (vGAT) and are inhibitory. Marker genes were characterized and selected for each cluster based on their expression level and uniqueness. Many of the marker genes were found to display a layer-specific pattern of expression,^38^ consistent with the anatomical feature of SC organization.^15^^,^^16^ In the current study, guided by this snRNA-seq atlas, we characterized 10 transgenic mouse lines to evaluate their usefulness to target specific sSC neuron types. We first characterized the Ntsr1-GN209-Cre and Grp-KH288-Cre mice, which have been widely used to target WFV and NFV cells, respectively. We then tested several other lines, including Grp-EGFP-Cre, Cart-IRES2-Cre-D, Slc1a3-CreER, and Vip-IRES-Cre, as well as vGluT2-IRES-Cre, for excitatory neuron types and vGAT-IRES-Cre mice, Cbln4-SN7-Cre, and Cbln4-IRES-mVenus-IRES-tdTomato for inhibitory neuron types. For each line, we examined the molecular identity of the labeled neurons in the sSC and evaluated whether they can target specific neuron types.

### Molecular identification of Cre+ cells in the sSC of Ntsr1-GN209-Cre mice

**Figure 1:**
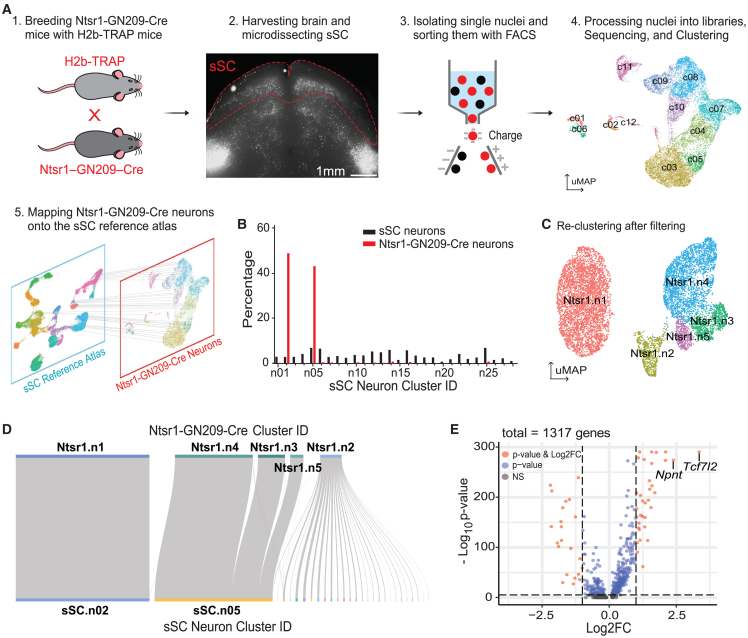
Molecular identification of superficial superior colliculus (sSC) cells in Ntsr1-GN209-Cre mice (A) Workflow of sequencing and annotating cells labeled in the Ntsr1-GN209-Cre mice. Ntsr1-GN209-cre mice were crossed with H2b-TRAP mice (A1) to label Cre-expressing cells with H2b-mCherry (A2). sSC was micro-dissected, and mCherry-expressing cells were isolated and sorted for sequencing (A3). Sorted cells were represented in a UMAP (uniform manifold approximation and projection) plot (A4), clustered by expression of highly variable genes and colored based on cluster identity, and compared to the published sSC molecular atlas (A5). Each dot in A4 and A5 represents a cell. Scale bar in A2: 1 mm. (B) Bar plot showing the percentage of sSC neurons in each of the 28 subtypes (black) and the percentage of Ntsr1-GN209-Cre+ neurons mapped to these subtypes (red). (C) UMAP plot illustrating the re-clustering results of Ntsr1-GN209-Cre neurons after filtering, referred to as Ntsr1 clusters. (D) Sankey plot visualizing the flow of each neuron from the five Ntsr1 clusters to 28 sSC neuron subtypes of the published reference atlas. The width of the gray line represents neuron numbers of each cluster. (E) Volcano plot depicting enriched genes expressed in sSC neuron cluster n02 and n05. Genes were colored based on thresholds: *p* value <1 × 10^−5^ and |log_2_FC| > 1 (FC, fold change). Pink indicated genes meeting both criteria, blue for *p* value only, and gray for neither.

Ntsr1-GN209-Cre mice have been used to label wide-field vertical (WFV) cells within the sSC.^12^^,^^14^^,^^29^^,^^30^^,^^36^ This is a BAC (bacterial artificial chromosome) transgenic line originated from the GENSAT project,^33^^,^^34^ where Cre recombinase expression is driven by exogenous promoters rather than endogenous gene regulation. To elucidate the molecular profile of Cre-expressing cells in the SC of these mice, we performed snRNA-seq analysis. Specifically, we crossed Ntsr1-GN209-Cre mice with Cre-dependent H2B-TRAP mice to label the histone H2B in the nuclei of Cre+ cells with mCherry fluorescent protein (Figure 1A1). This approach resulted in robust fluorescence expression in the stratum opticum (SO), deeper layers of the superior colliculus (dSC), and inferior colliculus (Figure 1A2). Subsequently, we isolated and sorted the labeled cells from the sSC of 11 adult male and female mice and profiled their genome-wide mRNA expression with snRNA-seq (Figure 1A3). After filtering out low-quality cells (see STAR Methods), we retained 16,854 cells for further analysis. Of these, neurons comprised 93.0% (15,667/16,854), whereas glial cells accounted for 3.4%, according to the expression of classical cell type marker genes (Figure S1B).

We then sought to determine how neurons in this dataset mapped onto the published sSC molecular atlas (Figure 1A5).^38^ To minimize technical bias that might force cells to map to the reference atlas, we used the glial cells as a control (Figure S1D). Given that the reference dataset was only composed of neurons, glial cells should not map to this reference atlas. When the maximum prediction score threshold was set at 0.9 (see STAR Methods for details), no glial cells were mapped to the reference datasets (Figure S1E). After applying this threshold, 60.2% of neurons remained (9,429/15,667). Importantly, the vast majority of these neurons (92.2%, 8,693/9,429) mapped to Clusters n02 and n05 in the reference sSC atlas (which we refer to as sSC clusters; Figure 1B). This mapping was not due to a higher composition of these two clusters in the reference atlas compared to the other clusters (Figure 1B) but instead reflected the existence of specific neuron types in Cre+ cells.

We next re-clustered the remaining 9,429 filtered neurons into five subtypes (Figure 1C), annotated as “Ntsr1.n1” through “Ntsr1.n5”. Four of the five clusters robustly expressed vGluT2 but little to no expression of vGAT, whereas the opposite was true for the cluster Ntsr1.n2 (Figure S1H). Neurons from Ntsr1.n2, 7.8% of the population, mapped to all 28 clusters in the reference atlas, possibly due to contaminations or artifacts in snRNA-seq. In contrast, all neurons from cluster Ntsr.n1 mapped to sSC.n02, and Ntsr1.n3, n4, and n5 merged into sSC.n05 (Figure 1D). Together, these results confirm that neurons with similar gene expressions consistently group together in different clustering methods and that there might exist potential subtypes within sSC.n05.

Next, we identified Nephronectin (*Npnt*) and Transcription Factor 7 Like 2 (*Tcf7l2*) as marker genes due to their enriched expression in sSC.n02 and sSC.n05 (Figure 1E). *Npnt* was selectively expressed within sSC.n02, whereas *Tcf7l2* was detected in both clusters but more robustly in sSC.05 (Figure S1H). Furthermore, we detected little to no expression of Neurotensin Receptor 1 (*Ntsr1*) in the reference atlas (Figure S1I), confirming that Cre expression in Ntsr1-GN209-Cre mice is not driven by endogenous *Ntsr1* expression.

### Verification of the Ntsr1-GN209-Cre line with selected marker genes

**Figure 2:**
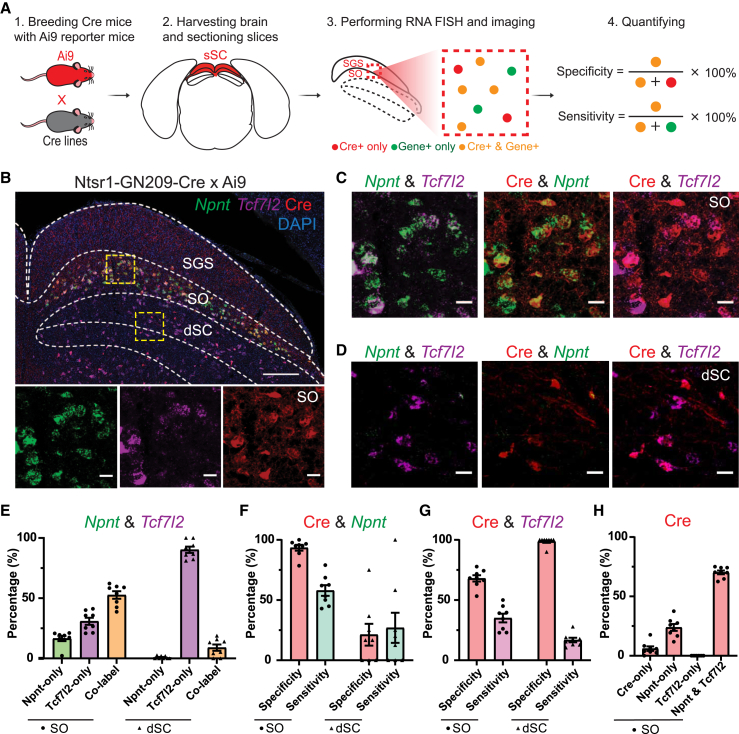
Verification of the Ntsr1-GN209-Cre line with selected marker genes (A) Workflow of evaluating specificity and sensitivity of a Cre mouse line driven by a selected marker gene. (B–D) RNA fluorescence *in situ* hybridization (FISH) of *Npnt* and *Tcf7l2* in the SC of Ntsr1-GN209-Cre x Ai9 mice. The area of yellow square in the SO (B, top) and deeper SC (dSC, B, top) is shown at a higher magnification with individual (B, bottom) and merged channels (C and D). Scale bars: 200 μm (B top) and 20 μm (B bottom, C, & D). (E) Percentage of neurons expressing *Npnt* only (green), *Tcf7l2* only (purple), or both (yellow) in the SO and dSC. Mean ± SEM. Each dot is from one SC image. *n* = 8 images, 3 mice. (F–G) Bar plot illustrating the specificity (pink) and sensitivity (green/purple) of Ntsr1-GN209-Cre line with *Npnt* (F) and *Tcf7l2* (G) expression in the SO (dot) and dSC (triangle). Each dot/triangle is from one SC image. Mean ± SEM. *n* = 8 images, 3 mice. (H) Bar plot showing the percentage of Cre+ neurons expressing Cre only, *Npnt* only (no *Tcf7l2*), *Tcf7l2* only (no *Npnt*), and both *Npnt* and *Tcf7l2* in the SO. Mean ± SEM. Each dot is from one SC image. *n* = 8 images, 3 mice.

To determine whether Cre-expressing SC neurons in Ntsr1-GN209-Cre mice express *Npnt* and/or *Tcf7l2*, we examined the co-expression of Cre and the two marker genes. We first crossed the Ntsr1-GN209-Cre mice with Cre-dependent reporter mice (Ai9) to induce Cre-mediated expression of tdTomato (Figures 2A.1 and 2A.2). We observed robust tdTomato expression in the SO sublayer of the sSC and sparse expression in the dSC (Figure 2B). Next, we performed RNA FISH to examine *Npnt* and *Tcf7l2* expression. Strong *Npnt* expression was detected in the SO, whereas *Tcf7l2* expression spanned from the lower SGS (stratum griseum superficiale) and SO to the dSC (Figure 2B). Co-expression of *Npnt* and *Tcf7l2* was detected in the SO, with approximately half of the cells (52.6% ± 3.1%) positive for either marker showing colocalization (Figures 2C and 2E). Among the remaining population, 16.5% ± 2.1% of the cells were only positive for *Npnt* and 30.8% ± 2.9% only for *Tcf7l2* (Figure 2E). In the dSC, only a minority of cells (8.9% ± 2.6%) positive for either marker displayed co-localization, and the majority (90.2% ± 2.6%) only expressed *Tcf7l2* (Figures 2D and 2E). These results are largely consistent with the expression patterns of *Npnt* and *Tcf7l2* in the reference atlas (Figure S1I), where they are preferentially expressed (but not uniquely) by cluster sSC.n02 and n05.

We then assessed the specificity and sensitivity of the Ntsr1-GN209-Cre line by calculating the percentage of cells co-expressing Cre and marker genes among all cells expressing Cre or marker genes (Figures 2A.3 and 2A.4). Within the SO, the vast majority of Cre+ cells (93.5% ± 2.4%) expressed *Npnt* (“specificity”, i.e., how specific is the Cre expression), and 58.0% ± 4.4% of *Npnt*+ cells expressed Cre (“sensitivity”, i.e., how efficient it is to label the marker gene expressing neurons, Figures 2C and 2F). For *Tcf7l2*, the specificity was 68.0% ± 2.6%, and the sensitivity was 35.1% ± 3.6% (Figures 2C and 2G). These results indicate that these mice exhibit a higher specificity and sensitivity in labeling *Npnt* expression in the SO than *Tcf7l2* expression. Furthermore, none of Cre+ cells were only positive for *Tcf7l2* but not *Npnt*, indicating that Cre-expressing cells in the SO are in fact from sSC.n02 (Figure 2H).

In the dSC, both specificity and sensitivity of Cre expression in labeling *Npnt* expression were low (specificity: 21.3% ± 9.0%; sensitivity: 27.0% ± 12.5%; Figures 2C and 2F). On the other hand, 98.8% ± 1.3% of Cre+ cells were positive for *Tcf7l2*, whereas 16.8% ± 1.9% of *Tcf7l2*+ cells were marked by Cre expression (Figures 2D and 2G). These results suggest that Cre-expression in these mice likely label different cell types in the dSC compared to the sSC. Together, we validated that Ntsr1-GN209-Cre line specifically labels a unique molecularly defined cell type in the SO expressing *Npnt* and marks another cluster in the dSC expressing *Tcf7l2*.

### Characterization of Grp-KH288-Cre and Grp-EGFP-Cre mice

**Figure 3:**
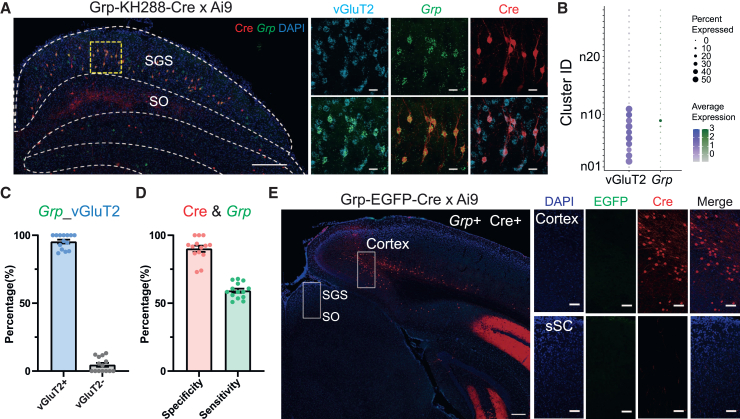
Characterization of two Grp-Cre mouse lines (A) RNA FISH of *Grp* in the SC of Grp-KH288-Cre x Ai9 mice. The area of yellow square on the left is shown at a higher magnification with individual (top) and merged (bottom) channels to the right. Scale bars: 200 μm (left) and 20 μm (right). (B) Dot plot showing that *Grp* is robustly expressed in sSC neuron cluster n09 (green), and cluster n09 is one of the excitatory clusters according to vGluT2 expression (blue). (C) Percentage of SGS *Grp*+ neurons that express vGluT2 (left) or not (right). Each dot is from one SC image. Mean ± SEM. *n* = 14 images, 4 mice. (D) Bar plot showing the specificity (pink) and sensitivity (green) of Grp-KH288-Cre line in the SGS. Each dot is from one SC image. Mean ± SEM. *n* = 14 images, 4 mice. (E) Cre expression in the SC and cortex of Grp-EGFP-Cre x Ai9 mice. The area of white rectangle on the left is shown at a higher magnification with individual and merged channels to the right. Scale bars: 200 μm (left) and 50 μm (right).

We next investigated the molecular identity of Cre-expressing sSC neurons in the Grp-KH288-Cre mice, which have been used to study narrow-field vertical cells (NFVs).^29^^,^^30^^,^^36^ Although the snRNA-seq approach as used in the previous section would be more unbiased, we opted for a simpler method by comparing the spatial location of the NFVs with specific marker gene expression from the reference atlas. Particularly, we crossed Grp-KH288-Cre mice with Ai9 mice to visualize Cre expression with tdTomato fluorescence. Cre+ neurons were detected mainly in the lower SGS with typical morphology of NFVs (Figure 3A). Based on the spatial information and the fact that NFVs are excitatory neurons, we screened the pattern of marker gene expression of the 28 sSC neuron subtypes and found that only Cluster n09 matched these conditions. Interestingly, this cluster displays an enriched expression of *Grp* (Gastrin Releasing Peptide) (Figure 3B), the same gene used to drive the Grp-KH288-Cre BAC transgenic line. This is different from the Ntsr1-GN209-Cre line tested in the previous section, where *Ntsr1* is not a cell type marker or even expressed in the sSC.

We performed RNA FISH of *Grp* and vGluT2 to test the hypothesis that Cre-expressing sSC cells in Grp-KH288-Cre mice may be related to Cluster n09. First, we confirmed that *Grp*+ cells are excitatory, with the vast majority (95.4% ± 1.4%) co-expressing vGluT2 (Figures 3B and 3C). Second, we detected a high specificity of this mouse line, in which 90.1% ± 2.3% of Cre+ cells were positive for *Grp* (Figures 3A and 3D). The sensitivity was relatively lower, with 59.2% ± 1.5% of *Grp*+ cells labeled by Cre expression (Figures 3A and 3D). These results show that almost all Cre+ cells in Grp-KH288-Cre mice expressed *Grp* (i.e., related to Cluster n09, although they may not label all neurons in this cluster).

In these mice, we noticed sparse tdTomato signal in the SO and dSC but no co-localization with *Grp* expression (Figure 3A). This indicated that Cre expression in these mice was not tightly correlated with endogenous *Grp* expression. This prompted us to characterize another Grp mouse line, Grp-EGFP-Cre. This is a knockin line that has an enhanced green fluorescent protein (EGFP) and Cre sequence inserted into the ATG start codon of the *Grp* gene, allowing EGFP to indicate Cre expression.^41^ Unfortunately, we detected no EGFP expression in the SC (Figure 3E). We also crossed them with Ai9, resulting in strong tdTomato expression in the cortex, but again no signal was detected in the SC (Figure 3E). This line is therefore not effective for accessing *Grp*+ cells in the SC. Furthermore, even for cortical expression, the pattern of Cre expression varied among individuals, even though they were all genotyped positive for *Cre* (Figures 3E and S2A). We then genotyped these mice with both *Cre* and *Grp* probes and found that some Cre+ mice were in fact negative for *Grp* (Figure S2A)*.* This result thus indicates a potential off-target of Cre insertion and the need for genotyping both probes in studies that may use Grp-EGFP-Cre mice for other brain regions.

### Characterization of mouse lines for other excitatory neuron types in the SC

**Figure 4:**
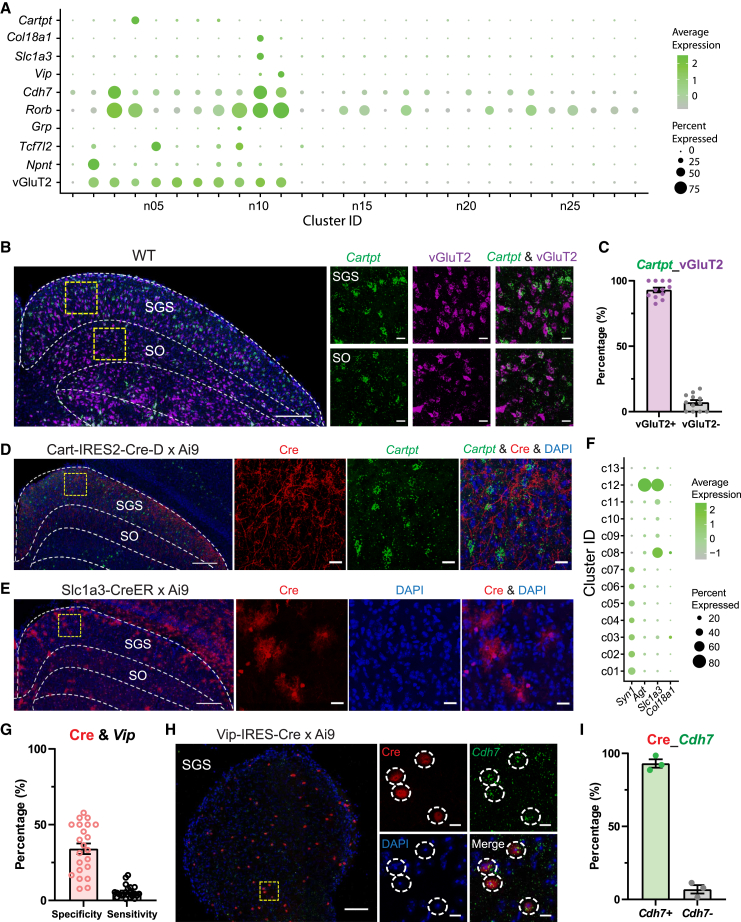
Genetic markers for sSC excitatory neuron clusters and characterization of three transgenic mouse lines driven by excitatory marker genes (A) Summary of marker gene expression for excitatory neurons (vGluT2) and subtypes (*Npnt*, *Tcf7l2*, *Grp*, *Cartpt*, *Slc1a3*, *Vip*, *Rorb*, *Cdh7*, and *Col18a1*). (B) RNA FISH of *Cartpt* and vGluT2 expression in the sSC. The area of yellow square on the left is shown at higher magnification with merged and individual channels to the right. Scale bars: 200 μm (left) and 20 μm (right). (C) Percentage of *Cartpt*+ cells co-expressing vGluT2 mRNA. Mean ± SEM. *n* = 12 images, 3 mice. (D) RNA FISH of *Cartpt* in the sSC of Cart-IRES2-Cre-D x Ai9 mice. The area of yellow square on the left is shown at a higher magnification with merged and individual channels to the right. Scale bars: 200 μm (left) and 20 μm (right). (E) Cre expression in the sSC of Slc1a3-CreER x Ai9 mice. The area of yellow square on the left is shown at a higher magnification to the right. Scale bars: 200 μm (left) and 20 μm (right). (F) The expression of *Syn1* (marker for neurons), *Agt* (marker for astrocytes), *Slc1a3*, and *Col18a1* in all sequenced sSC cells of the reference atlas.^38^ Cluster ID: c01-c07.Neurons, c08.Leptomeningeal cells, c09.Microglia, c10.Oligodendrocytes, c11.Oligodendrocyte progenitor cells, c12. Astrocytes, c13.Endothelial cells. (G) The specificity (pink) and sensitivity (black) of Vip-IRES-Cre line with *Vip* expression. Mean ± SEM. *n* = 3 mice. Calculated using previously published data.^38^ (H) RNA FISH of *Cdh7* expression at the SC surface (0–50 μm, horizontal view) of Vip-IRES-Cre x Ai9 mice. The area of yellow square on the left is shown at a higher magnification with merged and individual channels to the right. Scale bars: 100 μm (left) and 10 μm (right). (I) Percentage of Vip-IRES-Cre-expressing cells that co-express *Cdh7* in the surface of SC. Mean ± SEM. *n* = 3 mice.

The abovementioned results demonstrated that the Cre-expressing cells in the sSC of the two BAC lines in fact fall into distinct transcriptomic clusters, supporting a correspondence between molecular and morphological cell types. Stellate cells are another excitatory morphological type in the sSC, but no mouse line has been discovered to specifically label them. It was reported that a knockin line, Rorb-IRES-Cre, labels most stellate cells, approximately half of NFV cells, and some horizontal cells, but no WFVs.^30^ These results prompted us to investigate the expression of *Rorb* (RAR Related Orphan Receptor B) in the sSC atlas. We found that *Rorb* is strongly expressed in Clusters n03, n04, n10, n11, and n09 (the one labeled by Grp-KH288-Cre) and weakly expressed in many inhibitory clusters (Figure 4A). These results are consistent with the diverse composition of Rorb-IRES-Cre-labeled cells.^30^ Also consistent with the finding that Rorb-IRES-Cre mice do not label WFV cells, we observed low *Rorb* expression in Clusters n02 and 05 (Figure 4A), which are labeled in the Ntsr1-GN209-Cre mouse. Motivated by these results, we searched for transgenic mice that could target these additional Clusters (n03, n04, n10, and n11; Figure 4A), with the hope of using them in future studies to target specific types such as stellate cells. Specifically, we characterized Cart-IRES2-Cre-D line for Cluster n04, Slc1a3-CreER line for Cluster n10, and Vip-IRES-Cre line for Cluster n11.

#### Characterization of Cart-IRES2-Cre-D mice

*Cartpt* (Cocaine- And Amphetamine-Regulated Transcript Protein) is strongly and uniquely expressed in Cluster n04 and can be used as a marker gene for this cluster (Figure 4A). We first examined the transmitter expression of *Cartpt*-expressing cells by co-staining *Cartpt* and vGluT2 using RNA FISH. Indeed, the vast majority of *Cartpt*+ cells (93.0% ± 1.8%) expressed vGluT2 (Figures 4B and 4C), confirming the sequencing data. Interestingly, an existing line, the Cart-IRES2-Cre-D knockin mouse, has been used to label on-off direction selective retinal ganglion cells.^42^ Unfortunately, when checking the Cart-IRES2-Cre-D x Ai9 mice, we observed very few tdTomato-expressing cells in the sSC (Figure 4D). Instead, we detected abundant tdTomato-labeled processes in the sSC (Figure 4D), which were most likely retinal axons. We further used RNA FISH to stain Cre in the brain slices of Cart-IRES2-Cre-D mice. No Cre expression was observed in the sSC, but weak expression was seen in the cortex (Figure S2B). The minimal Cre expression explains the lack of tdTomato+ cells and indicates that this is not a useful line for labeling specific neuron types in the SC.

#### Characterization of Slc1a3-CreER mice

We next characterized Slc1a3-CreER mice for labeling *Slc1a3* (Solute Carrier Family 1 Member 3), a selected marker gene for Cluster n10 (Figure 4A). This BAC mouse line, also known as GLAST-CreER, expresses CreER under the control of *Slc1a3* promoter and has been used for neurodevelopmental studies.^43^^,^^44^ After crossing with Ai9 mice, tdTomato+ cells exhibited either a star-shaped morphology or appeared as small as cell nuclei stained by DAPI (Figure 4E). In other words, Slc1a3-CreER mice did not appear to label neurons in the SC. We further analyzed the sSC snRNA-seq data by examining all cell types without excluding non-neuronal types. The result showed that *Slc1a3* was strongly expressed in astrocytes and leptomeningeal cells and weakly expressed in Oligodendrocyte progenitor cells, oligodendrocytes, microglia, and neurons (Figure 4F). *Slc1a3* is therefore not a useful marker gene for studying neurons of Cluster n10.

Besides *Slc1a3*, *Col18a1* could be another marker gene for Cluster n10 according to the snRNA-seq data (Figure 4A). Promisingly, RNA FISH staining showed enriched *Col18a1* expression in the SGS (Figure S2C). It should be noted that *Col18a1* is also expressed in leptomeningeal cells according to snRNA-seq data (Figure 4F). Future experiments are needed to establish whether *Col18a1* can be used as an effective neuron type marker for this cluster.

#### Characterization of Vip-IRES-Cre mice

**Figure 5:**
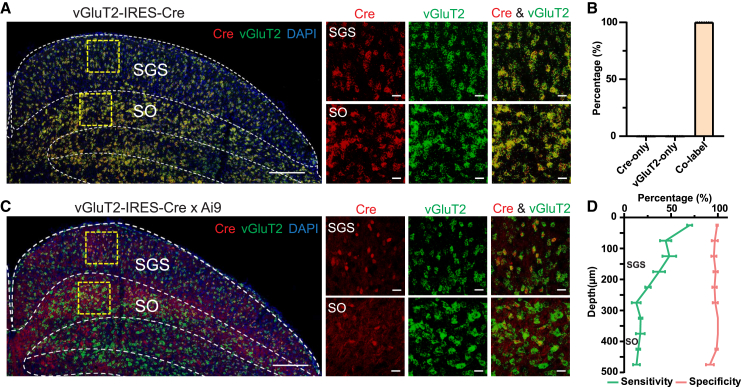
Characterization of vGluT2-IRES-Cre mice (A) RNA FISH of Cre and vGluT2 expression in the SC of vGluT2-IRES-Cre mice. The area of yellow square on the left is shown at a higher magnification with merged and individual channels to the right. Scale bars: 200 μm (left) and 20 μm (right). (B) Percentage of cells expressing only Cre, only vGluT2, and both. Mean ± SEM. *n* = 10 images, 3 mice. (C) RNA FISH of vGluT2 expression in the SC of vGluT2-IRES-Cre x Ai9 mice. The area of yellow square on the left is shown at a higher magnification with merged and individual channels to the right. Scale bars: 200 μm (left) and 20 μm (right). (D) Plot of specificity (pink) and sensitivity (green) of vGluT2-IRES-Cre line with vGluT2 expression along the SC depth. Mean ± SEM. *n* = 7 images, 4 mice.

*Vip* (Vasoactive Intestinal Peptide) is enriched in Cluster n11 (Figure 4A). In our previous paper,^38^ we identified that *Vip*+ neurons in the sSC express vGluT2. We also characterized Vip-IRES-Cre mouse line and found it faithfully labels *Vip*+ cells in the cortex but not in the SC. In the sSC, only a small portion of Cre+ cells (34.1% ± 3.5%) co-expressed *Vip* (specificity), and only 5.7% ± 0.9% of *Vip*-expressing cells were labeled by Cre (sensitivity, Figure 5G). Interestingly, even though these mice did not specifically target *Vip+* cells in the SC, we noticed that the vast majority of Cre+ cells near the SC surface (0–50 μm, 93.1% ± 2.9%) co-expressed *Cdh7* (Figures 5H and 5I). *Cdh7* was expressed in several excitatory clusters, including n11 (the *Vip+* cluster), n04, and n10, as well as weak expression in some inhibitory clusters according to the snRNA-seq data (Figure 4A). The exact reason underlying this expression pattern of Cre+ cells is unknown, but it may be useful information for future studies to investigate why this particular mouse line does not label *Vip+* cells in the SC.

#### Characterization of vGluT2-IRES-Cre mice

Given the overall disappointing results of the four lines of mice, we decided to characterize a commonly used line for studying excitatory cells in the SC, the vGluT2-IRES-Cre mice. In these mice, Cre expression is driven by vGluT2 and ideally can be used to label all excitatory sSC neurons (Figure 4A). Indeed, we observed a near-perfect overlap between Cre and vGluT2 expression (Figures 5A and 5B), suggesting that Cre expression faithfully labels vGluT2+ cells. However, after performing RNA FISH for vGluT2 on slices from vGluT2-IRES-Cre x Ai9 mice, it was challenging to identify clear soma with tdTomato signal, compared to the expression of vGluT2 in the sSC (Figure 5C). This difficulty was due to the widespread tdTomato expression across the entire sSC area. Therefore, we manually selected seven images with relatively discernible cellular shapes from 26 images of six mice to estimate the usefulness of this line. The specificity was very high (97.2% ± 0.6%, on average across the depth), whereas the sensitivity was much lower (30.1% ± 2.8%, on average across the depth; Figure 5D). The specificity remained high along the SC depth, contrasting with the decrease of sensitivity (Figure 5D). Therefore, crossing vGluT2-IRES-Cre with Ai9 does not appear to be an efficient way to label vGluT2+ neurons in the sSC.

### Characterization of mouse lines for inhibitory neuron subtypes in the SC

**Figure 6:**
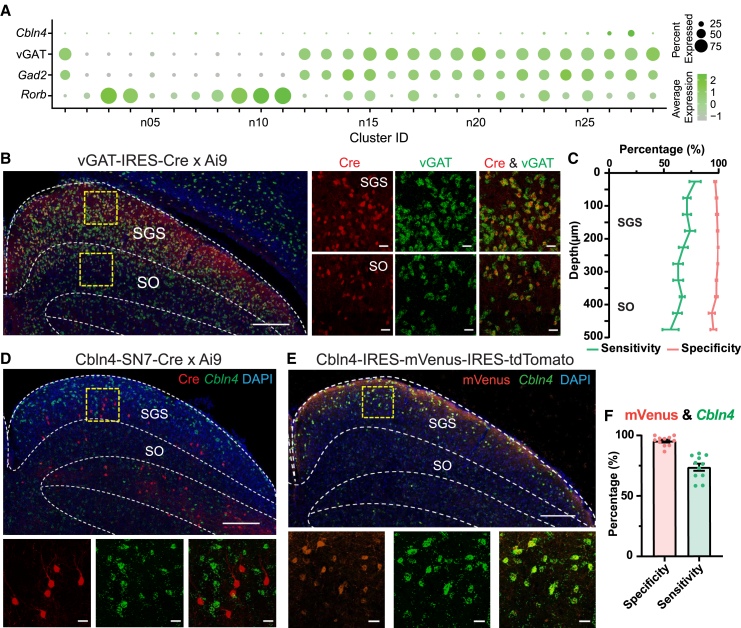
Genetic markers for sSC inhibitory neuron clusters and characterization of vGAT-IRES-Cre, Cbln4-SN7-Cre, and Cbln4-IRES-mVenus-IRES-tdTomato mice (A) Expression of marker genes for inhibitory neurons (Gad2, vGAT, *Cbln4*, *Rorb*). (B) RNA FISH of vGAT expression in the sSC of vGAT-IRES-Cre x Ai9 mice. The area of yellow square on the left is shown at a higher magnification with merged and individual channels to the right. Scale bars: 200 μm (left) and 20 μm (right). (C) Plot of specificity (pink) and sensitivity (green) of vGAT-IRES-Cre line with vGAT expression along the SC depth. Mean ± SEM. *n* = 8 images, 4 mice. (D) RNA FISH of *Cbln4* expression in the sSC of Cbln4-SN7-Cre x Ai9 mice. The area of yellow square on the top is shown at a higher magnification with merged and individual channels to the bottom. Scale bars: 200 μm (top) and 20 μm (bottom). (E) RNA FISH of *Cbln4* expression and immunohistochemistry staining of mVenus protein in the sSC of Cbln4-IRES-mVenus-IRES-tdTomato line. The area of yellow square on the top is shown at a higher magnification with merged and individual channels to the bottom. Scale bars: 200 μm (top) and 20 μm (bottom). (F) Bar plot showing the specificity (pink) and sensitivity (black) of Cbln4-IRES-mVenus-IRES-tdTomato line with *Cbln4* expression. Mean ± SEM. *n* = 11 images, 3 mice.

Previous studies used Gad2-Cre to mark horizontal cells, the only reported inhibitory morphological type in the sSC.^29^^,^^30^ However, *Gad2* (Glutamate Decarboxylase 2) expression is detected in all 18 inhibitory neuron subtypes in the sSC transcriptomic atlas, accompanied by vGAT expression (Figure 6A). This suggested a broad molecular diversity within inhibitory sSC neurons. We have therefore characterized vGAT-IRES-Cre mouse line, as well as two other lines for a subtype of inhibitory neurons.

#### Characterization of vGAT-IRES-Cre mice

To validate the fidelity of vGAT-IRES-Cre mice in labeling vGAT-expressing cells, we performed RNA FISH of vGAT in vGAT-IRES-Cre x Ai9 mice (Figure 6B). On average across depths, the majority (97.6% ± 0.8%) of Cre+ cells were found to be vGAT+ (specificity), and 67.4% ± 3.2% of vGAT expressing cells were labeled by tdTomato (sensitivity, Figure 6C). This result suggests that this mouse line could indeed specifically label vGAT+ cells in the sSC.

#### Characterization of Cbln4-SN7-Cre and Cbln4-IRES-mVenus-IRES-tdTomato mice

Cerebellin 4 Precursor (*Cbln4*) has recently been identified as a marker gene for Cluster n27 that labels inhibitory direction-selective cells in the sSC.^38^ To further investigate this cluster, we obtained two available *Cbln4* mouse lines. One line is Cbln4-SN7-Cre generated by the GENSAT project.^33^^,^^34^ We stained the *Cbln4* transcript in the SC of Cbln4-SN7-Cre x Ai9 mice, and the staining results demonstrated that Cre expression did not correlate with *Cbln4* expression (Figure 6D). Cre+ cells were sparsely distributed in the lower SGS, whereas the *Cbln4* transcript was predominantly enriched in the upper SGS. Our observation of Cre expression is consistent with the verification of Cbln4-SN7-Cre line by the GENSAT project (see website GENSAT: https://www.gensat.org/creGeneView.jsp?founder_id=91415&gene_id=1009).

The other line we tested is a *Cbln4* knockin line: Cbln4-IRES-mVenus-IRES-tdTomato, which carries conditional reporters mVenus and tdTomato.^45^ In these mice, *Cbln4+* cells are supposed to express *mVenus*, and upon Cre-mediated recombination, IRES-mVenus is deleted and the cells will express tdTomato. We used RNA FISH to detect *Cbln4* and immunohistochemistry to enhance mVenus signal in these mice, and we observed a great overlap between *Cbln4*+ cells and mVenus+ cells in the upper SGS (Figure 6E). Quantitatively, 95.3% ± 1.2% of mVenus-expressing cells co-expressed *Cbln4* (specificity), and 73.6% ± 3.0% of *Cbln4*-expressing cells were labeled by mVenus expression (sensitivity, Figure 6F). In other words, this knockin mice can specifically label *Cbln4* expression cells in the SC. However, the mVenus expression was not detectable in heterozygous mice and weak in homozygous mice, and thus needed antibody amplification. Consequently, these mice can be useful for experiments to specifically target *Cbln4+* cells in the sSC but that does not require direct visualization of these cells *in vivo*.

## Discussion

**Figure 7:**
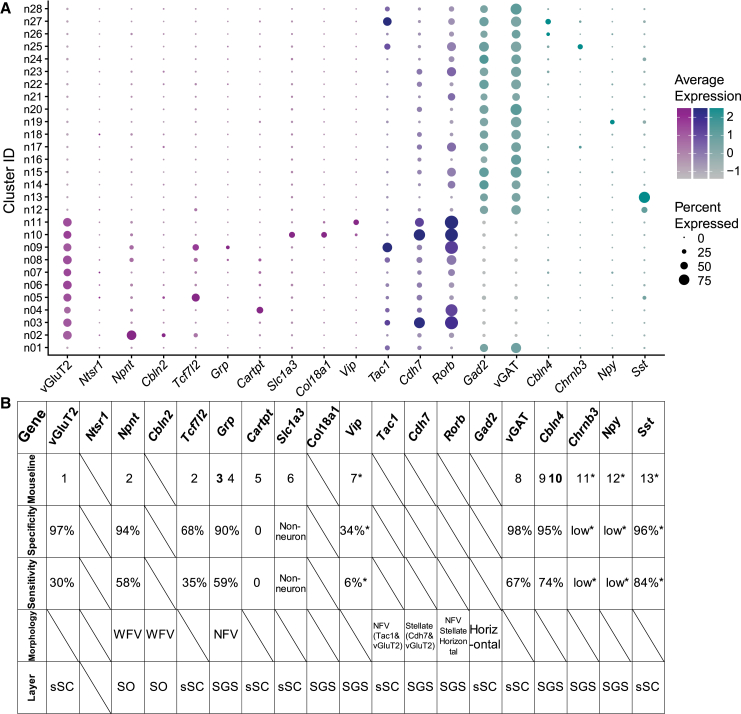
Summary of mouse lines for studying sSC cell types (A) Summary plot of marker gene expression across 28 neuron subtypes based on the reference atlas.^38^ Genes detected in excitatory neurons, magenta; in inhibitory neurons, cyan; and in both, blue. (B) Summary table of mouse lines corresponding to their marker genes, specificity and sensitivity, morphology, and layer distribution. Mouse lines (see strain details in STAR Methods): (1) vGluT2-IRES-Cre; (2) Ntsr1-GN209-Cre; (3) Grp-KH288-Cre; (4) Grp-EGFP-Cre; (5) Cart-IRES2-Cre-D; (6) Slc1a3-CreER; (7) Vip-IRES-Cre; (8) vGAT-IRES-Cre; (9) Cbln4-SN7-Cre; (10) Cbln4-IRES-mVenus-IRES-tdTomato; (11) Chrnb3-SM93-Cre; (12) Npy-RH26-Cre; (13) Sst-IRES-Cre. Results that were mentioned but not directly measured in this study were marked by asterisk.

The SC is one of the best studied brain structures due to its critical functions in sensorimotor processing, well-organized laminar structure, brain-wide connectivity, and conservation across species.^15^^,^^16^^,^^28^ Current knowledge about SC cell types is mostly from morphological and functional studies. Recent genomic studies have revealed a greater diversity of SC cell types than previously recognized and therefore provided an exciting opportunity to investigate the connectivity and function of these molecular cell types. In this study, as an important step toward performing such studies, we examined a number of transgenic mouse lines, which include GENSAT lines that have been used to target morphological cell types and other lines that potentially label specific molecular types according to the recently published molecular atlas of the mouse sSC.^38^ These results, as well as the expression profile of relevant genes, are summarized in Figure 7.

First, we found that the two GENSAT lines label distinct molecular cell types. Most Ntsr1-GN209-Cre+ neurons in the SO expressed *Npnt* (a specific marker for Cluster n02), and most Grp-KH288-Cre+ neurons in the lower SGS expressed *Grp* (a specific marker for Cluster n09; Figure 7). Cre-expressing cells in the Ntsr1-GN209-Cre mice also included another cluster (Cluster n05, expressing *Tcf7l2*; Figure 7). These cells were mostly seen in the deeper SC, likely reflecting contaminations during tissue collection. Such contaminations may even have included the inferior colliculus, where robust Cre expression and *Tcf7l2* expression were seen. Similarly, in the Grp-KH288-Cre mice, Cre-expressing cells were found in the SO and dSC, where *Grp* expression was rare (Figure 7). These results suggest that these GENSAT lines that were screened to label morphological cell types indeed target certain transcriptomic neuron types in the SC. However, extra care should be taken when using these mice, as Cre is also expressed outside the intended neurons.

Our findings are largely consistent with a recent study by Tsai et al. that combined transsynaptic tracing and single-cell sequencing (“Trans-Seq”) to classify SC neurons directly innervated by retinal ganglion cells.^14^ First, both studies identified that *Npnt,* as a marker gene of a neuron type, is expressed by Ntsr1-GN209-Cre-labeled cells. *Cbln2* (Cerebellin 2 Precursor) is another marker gene for the same cluster (sSC.n02) according to our atlas (Figure 7A), which is also consistent with other single-cell sequencing studies.^12^^,^^14^^,^^46^ Second, the Trans-Seq study showed that *Tac1* (Tachykinin Precursor 1) is highly expressed in the excitatory neurons that correspond to NFV cells (Grp-KH288-Cre mice) but also in inhibitory neuron types in the SC.^14^ This is consistent with our sSC snRNA-seq atlas, where *Tac1* is enriched in Cluster n09 as well as many other clusters.^38^ Here, we also identify *Grp* as a marker gene for this cluster (Figure 7A), providing a more specific access point to this neuron type. Third, the Trans-Seq study showed that Cre+ neurons in Rorb-IRES-Cre mice, which include stellate cells, express *Cdh7*.^14^ *Cdh7* is in fact expressed by many sSC clusters, but for excitatory neurons, it is mainly expressed by Cluster n03, n09, and n10 (Figure 7A). This suggests that one or more of these clusters likely correspond to stellate cells. Together, the consistent findings among these studies reveal a correspondence between cell types defined morphologically and transcriptomically.

We also examined a number of other mice in this study (Figures 7A and 7B). Our results verified that Cre expression targets specifically SC excitatory and inhibitory neurons in vGluT2-IRES-Cre and vGAT-IRES-Cre mice, respectively. Similarly, Cbln4-IRES-mVenus-IRES-tdTomato mice can be used to access *Cbln4+* neurons, which are inhibitory and display direction selective responses in the SC.^38^ On the other hand, *Grp* labels NFV cells that were found to be direction selective.^29^ Interestingly, *Cbln4* and *Grp* are expressed in the clusters n27 and n09, respectively, and *Tac1* is enriched in both according to the sSC atlas (Figure 7A). These findings suggest that *Tac1* may be a genetic marker for both excitatory and inhibitory direction selective cells. Furthermore, our sSC molecular atlas revealed that *Sst* (Somatostatin)-positive cells fall into two inhibitory clusters (n12 and n13, Figure 7A).^38^ Sst-IRES-Cre x Ai9 mice showed a remarkably high specificity (96.5% ± 1.4%, using previously published data^38^) and sensitivity (83.6% ± 1.5%,^38^; Figures 7A and 7B) in labeling *Sst+* cells. Our results have thus identified useful mouse lines for studying SC inhibitory neuron subtypes. In contrast and unfortunately, the results of the mouse lines tested for excitatory subtypes were all negative. The three tested knockin lines (Grp-EGFP-Cre for Cluster n09, Cartpt-IRES2-Cre-D for Cluster n04, and Vip-IRES-Cre for Cluster n11) failed to report the driver gene expression. This finding was very surprising because Cre is driven by endogenous gene expression in these mice, and they have been used successfully in other brain regions as mentioned in the Results.^38^^,^^41^^,^^42^ Importantly, all three driver genes were in fact expressed by sSC neurons that co-expressed vGluT2, which confirms the validity of the snRNA-seq data. Together, these results suggest a general low Cre efficiency or expression in the SC of these lines, which may reflect a difference of transcription process between the SC and other brain areas. Better mice models are therefore needed to target these SC neuron subtypes in future studies.

Finally, we screened two additional lines, Chrnb3-SM93-Cre and Npy-RH26-Cre, using GENSAT database (See strain details in key resources table). *Chrnb3* and *Npy* were chosen as marker genes for inhibitory Clusters n25 and n19, respectively (Figure 7A). *Chrnb3* shows an enriched expression in the SGS, whereas *Npy* is predominantly expressed in the lower SGS^38^ (and also see websites Allen Brain Atlas: https://mouse.brain-map.org/gene/show/72205, https://mouse.brain-map.org/gene/show/73806). However, a similar expression pattern was not seen in the sSC of either Cre line (see websites GENSAT: https://www.gensat.org/creGeneView.jsp?founder_id=91361&gene_id=724, https://www.gensat.org/creGeneView.jsp?founder_id=89513&gene_id=5). These mice are thus not useful for accessing *Chrnb3+* or *Npy+* cells in the SC.

### Limitations of the study

The main purpose of our study was to determine whether the tested Cre lines were able to effectively label neurons that express corresponding genes. However, depending on how unique a marker gene is expressed in a given cluster, being positive for the marker gene does not necessarily mean that they belong to that cluster. snRNAseq (as we did for Ntsr1-GN209-Cre mice) or FISH with additional marker genes (such as in spatial transcriptomics) would strengthen the identification of Cre+ neurons into specific transcriptomic clusters. It is important to note that we selected the marker genes based on their specific expression in certain clusters, layer distribution, and transmitter properties, which gives us the best genetic access point to individual clusters. Our results therefore provide valuable information regarding the usefulness of these mice despite this technical limitation.

Finally, we used Ai9 mice to report Cre expression. We observed consistent tdTomato expression in specific SC sublayers among individual samples for most lines. To evaluate the Cre expression in these mice, we used two complementary criteria—specificity and sensitivity. For useful lines, the specificity is very high (∼90%) and sensitivity varies between ∼60% and ∼80% (Figure 7B). The observation that sensitivity is always lower than specificity may reflect a potential bias of our method to evaluate mouse lines. The expression of tdTomato in Ai9 mice requires sufficient Cre activity for the fluorescence to be detectable. In contrast, RNAScope is likely more sensitive in detecting gene transcripts.^47^ As a result, the quantification of tdTomato fluorescence may underestimate the number of Cre-expressing cells. On the other hand, overexpressed and widespread tdTomato labeling have also been observed, and the strong background makes it very difficult or even impossible to determine whether a particular neuron is indeed tdTomato positive. Alternative tools, such as Cre-dependent viruses, may be necessary for accurate reporting of Cre-expressing cells. However, these methods also have limitations, such as the need for surgical procedures, non-uniform expression, tissue-specific variability, promoter affinity issues, and potentially lower specificity.^48^ Therefore, it is crucial to select the most appropriate method based on the experimental requirements and to carefully evaluate both specificity and sensitivity for each method and mouse line before proceeding.

## Resource availability

### Lead contact

Further information and requests for experimental data should be directed to and be fulfilled by the lead contact, Jianhua Cang (cang@virginia.edu).

### Materials availability

This study did not generate any unique reagents.

### Data and code availability

• The raw and processed snRNA-seq data reported in this paper have been deposited at GEO website and publicly available as of the date of publication. Accession number is listed in the key resources table.
• R code used in the current study has been deposited at Zenodo and publicly available as of the date of publication. Access link is listed in the key resources table.
• Any additional information required to reanalyze the data reported in this paper is available from the lead contact upon request.

## Acknowledgments

We thank Dr. Martha Bickford for providing the Grp-KH288-Cre mice; Dr. John Campbell for providing the H2b-TRAP and Grp-EGFP-Cre mice; and Dr. Wei Wei for providing Cart-IRES2-Cre-D brain tissues. This work was supported by US 10.13039/100000002NIH grants (EY026286 and EY020950) and 10.13039/100019530Jefferson Scholars Foundation to J.C.

## Author contributions

J.C., C.C., and Y.L. designed the experiments; C.C. performed data analysis on the sSC reference atlas with assistance from Y.L.; Y.L. performed snRNA-seq experiments, data analysis, and RNA FISH on Ntsr1-GN209-Cre mice; C.C. collected brain tissue, did RNA FISH, and performed imaging on all other mouse lines, with additional assistance from Y.L. on Slc1a3-CreER line; C.C. performed all quantifications; C.C., Y.L., and J.C. wrote the manuscript.

## Declaration of interests

The authors declare no competing interests.

## STAR★Methods

### Key resources table

**Table:** 

REAGENT or RESOURCE	SOURCE	IDENTIFIER
**Antibodies**		

Chicken Anti-GFP	Aves Labs	Cat# GFP-1020; RRID: AB_2307313

**Chemicals, peptides, and recombinant proteins**		

TSA Plus Fluorescein Reagent	Akoya Biosciences	Cat #: NEL741001KT
TSA Plus Cy3 Reagent	Akoya Biosciences	Cat #: NEL744001KT
TSA Plus Cy5 Reagent	Akoya Biosciences	Cat #: NEL745001KT
RNAprotect tissue reagent	QIAGEN	Cat #: 76106

**Critical commercial assays**		

Chromium Next GEM Single Cell 3′ GEM, Library & Gel Bead Kit v3.1, 4 rxns	10X Genomics	Cat #: 1000128
Chromium Next GEM Chip G Single Cell Kit, 16 rxns	10X Genomics	Cat #: 1000127
Single Index Kit T Set A, 96 rxns	10X Genomics	Cat #: 1000213
Invitrogen Qubit 1X dsDNA HS Assay Kit	ThermoFisher Scientific	Cat #: Q33231
RNAscope® Multiplex Fluorescent Reagent Kit v2	Advanced Cell Diagnostics	Cat #: 323100
RNAscope HiPlex12 Reagent Kit v2 (488, 550, 650)	Advanced Cell Diagnostics	Cat #: 324419
NextSeq 2000 P3 XLEAP-SBS Reagent Kit (100 cycles)	Illumina	Cat #: 20100990

**Deposited data**		

Seurat code used for cell clustering and making figures	This paper	https://zenodo.org/records/14113265
Raw and processed RNA seq data	This paper	GSE287867

**Experimental models: Organisms/strains**		

Mouse: vGluT2-IRES-Cre: B6J.129S6(FVB)-*Slc17a6*^*tm2(cre)Lowl*^/MwarJ	The Jackson Laboratory, JAX	Strain#:028863; RRID: IMSR_JAX:028863
Mouse: Ntsr1-GN209-Cre: Tg(Ntsr1-Cre)GN209Gsat	The Mutant Mouse Resource and Research Center, MMRRC	MMRRC:030780-UCD
Mouse: Grp-KH288-Cre: Tg(Grp-cre)KH288Gsat/Mmucd	The Mutant Mouse Resource and Research Center, MMRRC	RRID:MMRRC_031183-UCD
Mouse: Grp-EGFP-Cre: B6; 129S-*Grp*^*tm1.1(EGFP/cre)Zfc*^/J	The Jackson Laboratory, JAX	Strain #:033174; RRID:IMSR_JAX:033174
Mouse: Cart-IRES2-Cre-D: B6; 129S-*Cartpt*^*tm1.1(cre)Hze*^/J	The Jackson Laboratory, JAX	Strain #:028533; RRID:IMSR_JAX:028533
Mouse: Slc1a3-CreER: Tg(Slc1a3-cre/ERT)1Nat/J	The Jackson Laboratory, JAX	Strain #:012586; RRID:IMSR_JAX:012586
Mouse: Vip-IRES-Cre: *Vip*^*tm1(cre)Zjh*^/J	The Jackson Laboratory, JAX	Strain #010908 RRID: IMSR_JAX:010908
Mouse: vGAT-IRES-Cre: B6J.129S6(FVB)-*Slc32a1*^*tm2(cre)Lowl*^/MwarJ	The Jackson Laboratory, JAX	Strain #:028862; RRID:IMSR_JAX:028862
Mouse: Cbln4-SN7-Cre: Tg(Cbln4-cre)SN7Gsat/Mmucd	The Mutant Mouse Resource and Research Center, MMRRC	RRID:MMRRC_036639-UCD
Mouse: Cbln4-IRES-mVenus-IRES-tdTomato: *Cbln4*^*tm1.1Sud*^/J	The Jackson Laboratory, JAX	Strain #:032950; RRID:IMSR_JAX:032950
Mouse: Chrnb3-SM93-Cre: Tg(Chrnb3-cre)SM93Gsat/Mmucd	The Mutant Mouse Resource and Research Center, MMRRC	RRID:MMRRC_036469-UCD
Mouse: Npy-RN26-Cre: Tg(Npy-cre)RH26Gsat/Mmucd	The Mutant Mouse Resource and Research Center, MMRRC	RRID:MMRRC_034810-UCD
Mouse: Sst-IRES-Cre: *Sst*^*tm2.1(cre)Zjh*^/J	The Jackson Laboratory, JAX	Strain #013044 RRID: IMSR_JAX:013044
Mouse: Ai9: B6.Cg-*Gt(ROSA)26Sor*^*tm9(CAG-tdTomato)Hze*^/J	The Jackson Laboratory, JAX	Strain #007909; RRID: IMSR_JAX: 007909
Mouse: H2b-TRAP: B6.Cg-*Gt(ROSA)26Sor*^*tm1(CAG-HIST1H2BJ/mCherry,-EGFP/Rpl10a)Evdr*^/J	The Jackson Laboratory, JAX	Strain # 029789; RRID: IMSR_JAX: 029789
Mouse: C57Bl/6j: C57BL/6J	The Jackson Laboratory, JAX	Strain #000664, RRID:IMSR_JAX: 000664

**Software and algorithms**		

R	R version 4.3.1	https://cran.r-project.org/bin/windows/base/old/4.0.0/
Seurat v4.3.0	Hao et al.^49^	https://satijalab.org/seurat/
Cell ranger 5.0.0	10X Genomics	https://www.10xgenomics.com/
Zen 3.4 software (blue edition)	ZEISS	https://www.zeiss.com/microscopy/en/products/software/zeiss-zen.html
Illustrator	Adobe	https://www.adobe.com; RRID: SCR_010279
Prism	GraphPad	https://www.graphpad.com/scientific-software/prism/

### Experimental model and study participant details

#### Animals

This study used mice from strains indicated in key resources table of either sex between 6 weeks and 10 months. We used 8 female and 3 male Ntsr1-GN209-cre x H2b-TRAP mice for snRNA-seq of the labeled cells in the superficial Superior Colliculus (sSC). We crossed following strains with Ai9 to label Cre-expressing cells with tdTomato: vGluT2-IRES-Cre, Tg(Ntsr1-cre)GN209Gsat/Mmucd, Tg(Grp-Cre)KH288Gsat/Mmucd, Grp-EGFP-Cre, Cart-IRES2-Cre-D, Slc1a3-CreER, Vip-IRES-Cre, vGAT-IRES-Cre, Tg(Cbln4-Cre)SN7Gsat/Mmucd, Sst-IRES-Cre. Three Tg(Ntsr1-cre)GN209Gsat/Mmucd x Ai9 mice were used for RNA fluorescent *in situ* hybridization (FISH) of *Npnt* and *Tcf7l2*. Four Tg(Grp-Cre)KH288Gsat/Mmucd x Ai9 mice were used for RNA FISH of *Grp* and *vGluT2*. Three vGluT2-IRES-Cre mice were used for RNA FISH of *Cre* and *vGluT2.* Six vGluT2-IRES-Cre x Ai9 mice were used for RNA FISH of *vGluT2.* Three Vip-IRES-Cre x Ai9 mice were used for RNA FISH of *Cdh7.* One Cart-IRES2-Cre-D x Ai9 mouse and one Cart-IRES2-Cre-D mouse were used for RNA FISH of *Cartpt* and Cre. Four vGAT-IRES-Cre x Ai9 mice were used for RNA FISH of vGAT. Three Cbln4-IRES-mVenus-IRES-tdTomato and three Tg(Cbln4-Cre)SN7Gsat/Mmucd x Ai9 mice were used for RNA FISH of *Cbln4*. Four C57BL/6J mice for RNA FISH of *Cartpt* and vGluT2. One C57BL/6J mouse was used for RNA FISH of *Col18a1*. All mice were kept on a 12 h/12 h light/dark cycle in the animal room. Two to five animals were housed per cage. All experimental procedures were approved by the University of Virginia Institutional Animal Care and Use Committee.

### Method details

#### Single-nucleus RNA-sequencing

Brains were harvested after rapid decapitation, cooled in ice-cold 1x PBS for 1.5min, placed into a chilled stainless steel brain matrix, and then sectioned coronally into 1mm thick slices containing the sSC (Bregma -3.0 mm to -5.0 mm). The slices were immediately placed in RNAprotect reagent at 4°C overnight to stabilize RNA. The next day, the sSC was visualized under a fluorescent dissecting microscope, micro-dissected approximately by knife cuts, and processed into single-nuclei suspension using a previously published detergent-mechanical lysis protocol. Briefly, we homogenized the sSC in lysis buffer and separated nuclei from cellular debris using density gradient centrifugation. After resuspending the nuclei pellet, DRAQ5 was added to the resuspension, and nuclei positive for mCherry were filtered using the Becton Dickinson Influx Cell Sorter. Specifically, sorting was performed with SCYM (ASCP)-certified technical assistance at the University of Virginia Flow Cytometry Core, using a 70 μm nozzle in purity mode. To distinguish nuclei from non-nucleated debris, events were gated based on high DRAQ5 fluorescence intensity, using a 650 nm excitation laser and a 670/30 nm collection filter. Nuclei were further gated by forward scatter area vs. side scatter area to exclude large aggregates, followed by forward scatter area vs. forward scatter height to select for singlets. Finally, nuclei with high relative mCherry and GFP fluorescence (indicating nucleolar tagging by Cre-induced EGFP) were selected using a 561 nm excitation laser with a 610/20 nm collection filter for mCherry and a 488 nm excitation laser with a 530/30 nm collection filter for GFP.

Next, we split the sorted nuclei into 2 technical replicates, used 10X Chromium Next GEM 3’ reagent kit v3.1 according to the manufacturer’s protocol (user guide revision D) to capture each nucleus’s poly-adenylated RNA in a droplet, process it into cDNA sequencing libraries, and sequence them by Illumina Next-Seq 2000 to an average of 24,108 mean reads per cell. Approximately 16,500 nuclei per lane were loaded into two lanes (batches) of the same 10X chip. The sequencing reads from these two batches were demultiplexed with the 10X Cell Ranger pipeline (version 5.0.0), aligned to a custom “pre-mRNA” reference genome created from the mouse reference genome RGCm38.98, and merged to generate a feature-barcode matrix.

#### snRNA-seq data processing and analysis

snRNA-seq feature-barcode matrices were analyzed in R (version 4.3.1) using the Seurat package (version 4.3.0).^49^ Initially, we filtered out genes detected in fewer than three cells and cells in which we detected fewer than 200 genes. Then we filtered cells based on their sample-specific distribution of quality metrics: fewer than 5,000 detected genes, fewer than 15,000 detected unique molecular identifiers (nUMIs), and less than 3% of reads mapping to mitochondrial genes. Subsequently, we log-normalized the data with a scale factor of 10,000; selected the 2,000 most variable genes; scaled the data using all detected genes; performed Principal Component Analysis (PCA) to linearly reduce the dimensionality of the highly variable gene set; clustered the cells using the Louvain algorithm, based on Euclidean distance in the PCA space comprising the first 12 PCs and with a resolution value of 0.3; and performed non-linear dimensionality reduction by Uniform Manifold Approximation and Projection (UMAP)^50^ for visualization in two dimensions. Cluster relatedness in PCA space was illustrated with dendrograms using the BuildClusterTree() function in Seurat.

We then checked each cluster’s expression of cell type marker genes. Twelve distinct cell clusters were identified from the Ntsr1-GN209-cre x H2b-TRAP mice and annotated based on their expression of classical cell type marker genes: neurons (*Syn1*+, *Rbfox3*+), excitatory neurons (*Slc17a6*/vGluT2+), inhibitory neurons (*Slc32a1*/vGAT+), astrocytes (*Agt*+), and oligodendrocytes (*Mbp*+, Figure S1A). Neurons also exhibited a greater number of detected genes and unique molecular identifiers compared to glial cells. Based on cluster-level and cell-level co-expression of these cell type marker genes, we removed the suspect clusters potentially representing cell doublets. After that, we re-clustered cells from the neuronal group, including the steps of feature selection, PCA, and clustering with the top 10 PCs and resolution setting of 0.2.

We identified neuron cell types using the FindTransferAnchors() and TransferData() functions in Seurat (label transfer annotation) to project neurons (query dataset) onto a published sSC neuron molecular atlas (reference dataset). We used glial cells as a control for setting maximum prediction score at 0.9, such that none of them were mapped to the reference atlas. By using different prediction scores for neuronal mapping process, we found that the labeled neurons were mainly mapped to two clusters of the reference atlas, Cluster n02 and n05. The proportion of neurons mapped to these two clusters increased over the maximum prediction score (Figure S1F). Specifically, as the maximum prediction score increased, the percentage of neurons mapped to Cluster n02 (sSC.n02) rose, while the percentage of neurons mapped to Cluster n05 (sSC.n05) declined (Figure S1G). The latter percentage was smaller compared to the overall percentage of filtered neurons. Then we filtered neurons with cutoff 0.9 and re-clustered them with the top 7 PCs and a resolution setting of 0.2. After this strict quality control process, we used the gvisSankey() function to uncover the mapping relationship of labeled neurons in Ntsr1-GN209-cre mice to the published sSC atlas.

#### Tamoxifen-induced Cre expression

To induce Cre recombinase expression in adult Slc1a3-CreER mice, tamoxifen was administered via intraperitoneal injection at a dose of 200 mg/kg body weight for five consecutive days. Tamoxifen (Sigma, Cat# 10540-29-1) was freshly prepared by first dissolving it in 200-proof ethanol, followed by dilution in corn oil (Sigma, Cat# 8001-30-7) to a final concentration of 2 mg/ml. The solution was vortexed thoroughly to ensure complete dissolution.

#### Perfusion and slicing

For coronal slicing, animals were deeply anesthetized with an intraperitoneal injection of a lethal dose of Euthasol. Subsequently, they were perfused with 1x PBS followed by 4% paraformaldehyde (PFA). After perfusion, brains were separated from attached tissues and the skull. They were then fixed in 4% PFA at 4°C overnight. Fixed brains underwent dehydration in 30% sucrose at 4°C for 3 days. Once sunk to the bottom of the container, brains were embedded in O.C.T. compound, quick-frozen in a -80°C refrigerator, and sliced into 40 μm sections using a cryostat (Leica CM1950) at -20°C.

For horizontal slicing, the skull was kept intact with the brain after perfusion and fixed in 4% PFA at 4°C overnight. Following fixation, the skull was removed and the brain was placed in 5% agarose with the SC facing upward. The SC was then sliced horizontally into 40/50 μm sections using a vibratome (Leica VT1000S).

After slicing in either orientation, brain sections were collected and stored in cryoprotectant at -20°C for subsequent staining procedures.

#### RNA fluorescence *in situ* hybridization (FISH) and immunohistochemistry

The RNA FISH procedures followed methods outlined in our previous publication.^38^ Depending on the probes used, either MultiPlex or HiPlex assays were conducted (Table S1). Brain sections stored in cryoprotectant were initially washed twice in 1x phosphate-buffered saline (PBS, pH 7.2) for 10 minutes and mounted on Fisher slides (Cat. 12-550-15) overnight. The following day, staining of brain sections was carried out using RNAscope Multiplex v2 (Cat. 323100) or HiPlex v2 reagent kit (Cat. 324419), as per the respective assay protocols.^51^ Briefly, slide-mounted slices were rinsed with PBS, dehydrated sequentially in 50%, 70%, and 100% ethanol, circled with a hydrophobic barrier, treated with protease IV, and then incubated with targeted probes at 40°C for 2 hours. Subsequently, they were subjected to Amp 1-3 treatments. Probes utilized included *Npnt*, *Tcf7l2*, *Grp*, *Slc17a6 (*vGluT2*)*, *Vip*, *Cdh7*, *Cartpt, Col18a1, Slc32a1 (*vGAT*)*, and *Cbln4*. For the Multiplex procedure, hydrogen peroxide was added to floating brain sections for 10 minutes to quench endogenous peroxidase activity before mounting on slides. HRP-C1/C2/C3, dye FITC/Cy3/Cy5, and an HRP blocker were applied to bind Amp3 and trigger the fluorescence. In contrast, in the HiPlex protocol, corresponding fluorophores were directly added onto Amp3 to visualize targeted gene expression.

In Cbln4-IRES-mVenus-IRES-tdTomato mice, immunohistochemistry was employed to enhance the mVenus signal following RNA FISH. Post FISH, brain sections underwent two washes with PBST (0.5% Triton X-100 in PBS) for 5 minutes each. Sections were then incubated in blocking buffer (0.5% Triton X-100 and 5% normal donkey serum, NDS in PBS) for 1 hour at room temperature. The primary antibody (anti-GFP-chicken, diluted 1:300 in blocking buffer) was applied to brain sections overnight at 4°C. The following day, sections were incubated with a secondary antibody (anti-chicken 488, diluted 1:250 in PBS) for 2 hours at room temperature. Finally, DAPI was used as a nuclear counterstain, and fluorescence images were acquired using a confocal microscope (Zeiss LSM 800) with objectives ranging from 10X/0.45 to 20X/0.80 Plan Apo.

### Quantification and statistical analysis

The quantification of probe-positive and tdTomato+/mVenus+ cells was performed using Zen 3.4 software (blue edition), based on puncta density for FISH and fluorescence intensity for tdTomato/mVenus. First, the regions of interest were selected as follows: (1) vGluT2-IRES-Cre mice and vGAT-IRES-Cre mice, a 500 μm ∗ 250 μm rectangle was drawn from the SC surface. The rectangle was divided into 10 subregions, each 50 μm deep along the SC depth. (2) Nstr1-GN209-Cre, Grp-KH288-Cre and Cbln4-IRES-mVenus-IRES-tdTomato mice, two squares (Ntsr1: 30702.57 μm^2^; Grp & Cbln4: 30822.98 μm^2^) was drawn in the SGS or SO in the middle and lateral area of one SC image. Values for each SC image were averaged from these two squares. (3) *Cdh7* expression in Vip-IRES-Cre mice, all tdTomato+ cells in the first horizontal section of SC were counted. (4) Co-staining of *Cartpt* and vGluT2 in WT mice as well as co-staining of Cre and vGluT2 in vGluT2-IRES-Cre mice, a square of 30822.98 μm^2^ was randomly drawn in the sSC of each SC image.

Second, positive neurons were manually labeled by the Points Events function in the Zen software for each region. Probe-positive cells were selected when a group of puncta showed a neuronal shape, and the puncta density was higher than the background noise. Similarly, tdTomato+/mVenus+ cells were marked when the fluorescence intensity of a cell was higher than the background. When both conditions were met and they shared similar size and shape, the cells were considered as colocalized cells.

At last, specificity and sensitivity were calculated as follows: specificity = Number of cells positive to both transcript and Cre / Number of Cre+ cells; sensitivity = Number of cells positive to both transcript and Cre / Number of transcript+ cells. (Cbln4-IRES-mVenus-IRES-tdTomato mice, mVenus was used instead of Cre).

All values reported were mean ± SEM, and number of replicates were listed in figure legends.
