# Supplementary material for: Accessing genetically defined cell types in the superior colliculus with transgenic mouse lines
Source: iScience. 2025 Mar 11;28(4):112194. doi: 10.1016/j.isci.2025.112194 (PMC11982483; doi:10.1016/j.isci.2025.112194)
Supplement: Document S1. Figures S1 and S2 and Table S1 [file mmc1.pdf]

iScience, Volume *28*

Supplemental information

Accessing genetically defined cell types in the superior colliculus with transgenic mouse lines

Chen Chen, Yuanming Liu, and Jianhua Cang

Supplemental Figures and Table

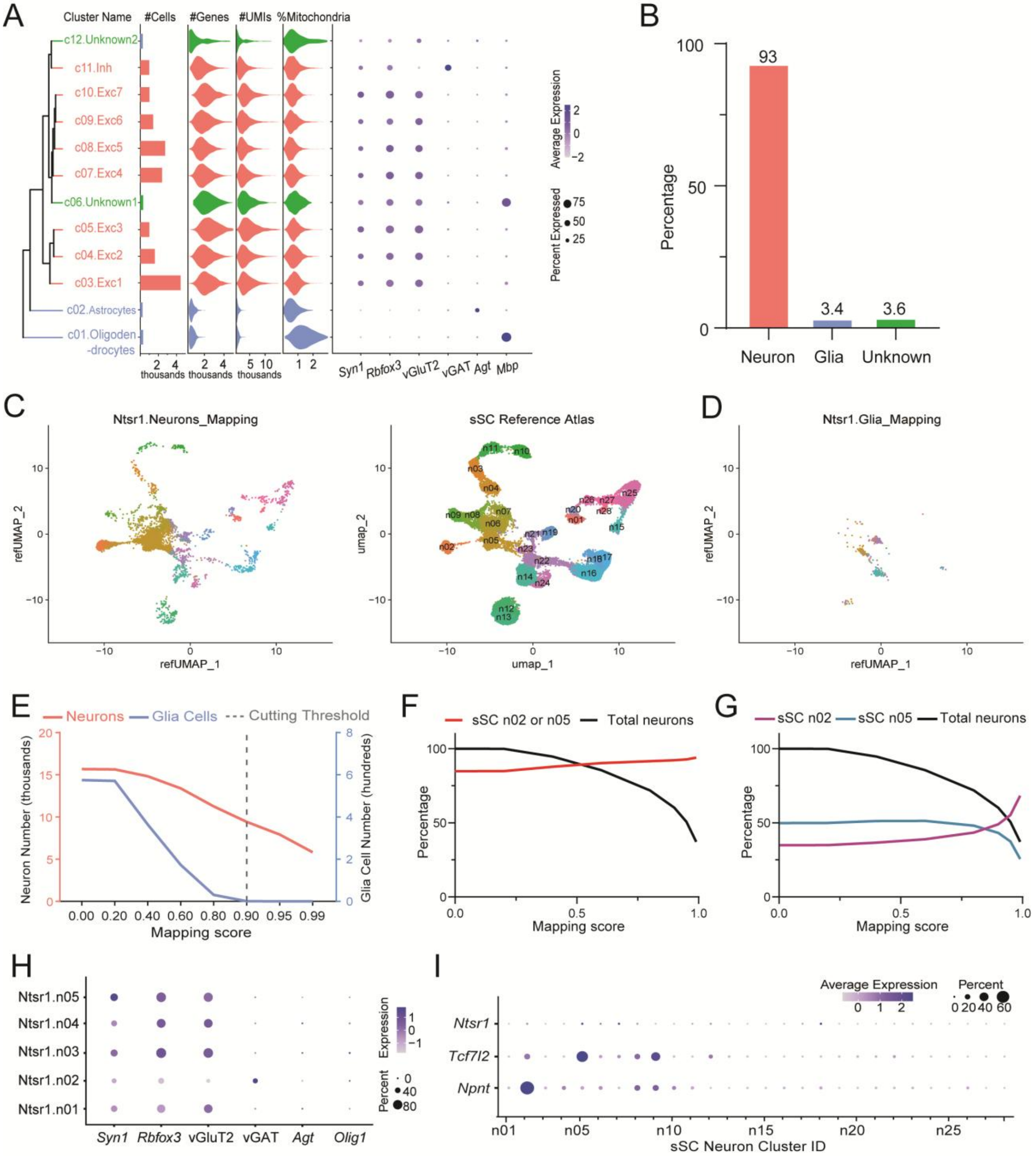

Figure S1. Additional information of molecular identification of sSC cells in Ntsr1-GN209-Cre mice, Related to Figure 1

(A) Dendrogram showing molecular relatedness of clusters of Cre positive cells, followed by cluster names; number of cells per cluster; number of genes detected per cluster; number of unique molecular identifiers (UMIs) per cluster; percentage of mitochondria gene expression per cluster; and plot illustrating average expression and percentage of cells expressing the selected marker genes for each cell type.

(B) Bar plot showing the percentage of neurons, glia, and unknown cells in the dataset.

(C) UMAP plot visualizing the Ntsr1-GN209-Cre neurons (left) mapped to the reference atlas[38] (right).

(D) UMAP plot illustrating the Ntsr1-GN209-Cre glia mapped to the reference atlas.

(E) Line graph showing the number of neurons (red, left axis) and glia (blue, right axis) as a function of the maximum prediction score after label transfer annotation.

(F) The black line plots the percentage of number of neurons after filtering (black) relative to the pre-filtering number, as a function of the maximum prediction score. The red line plots percentage of neurons mapped to clusters n02 or n05, relative to the total number of neurons after filtering, as a function of the maximum prediction score.

(G) Percentage of neurons mapped to Cluster n02 neurons (red) and n05 neurons (blue), relative to the total number of neurons plotted against the maximum prediction score. The black line is the same as the one in (F) for reference.

(H) Dot plot illustrating average expression and percentage of cells expressing the selected marker genes for each Ntsr1-GN209-Cre cluster.

(I) Dot plot illustrating expression of *Npnt*, *Tcf7l2* and *Ntsr1* across 28 clusters in the reference atlas.

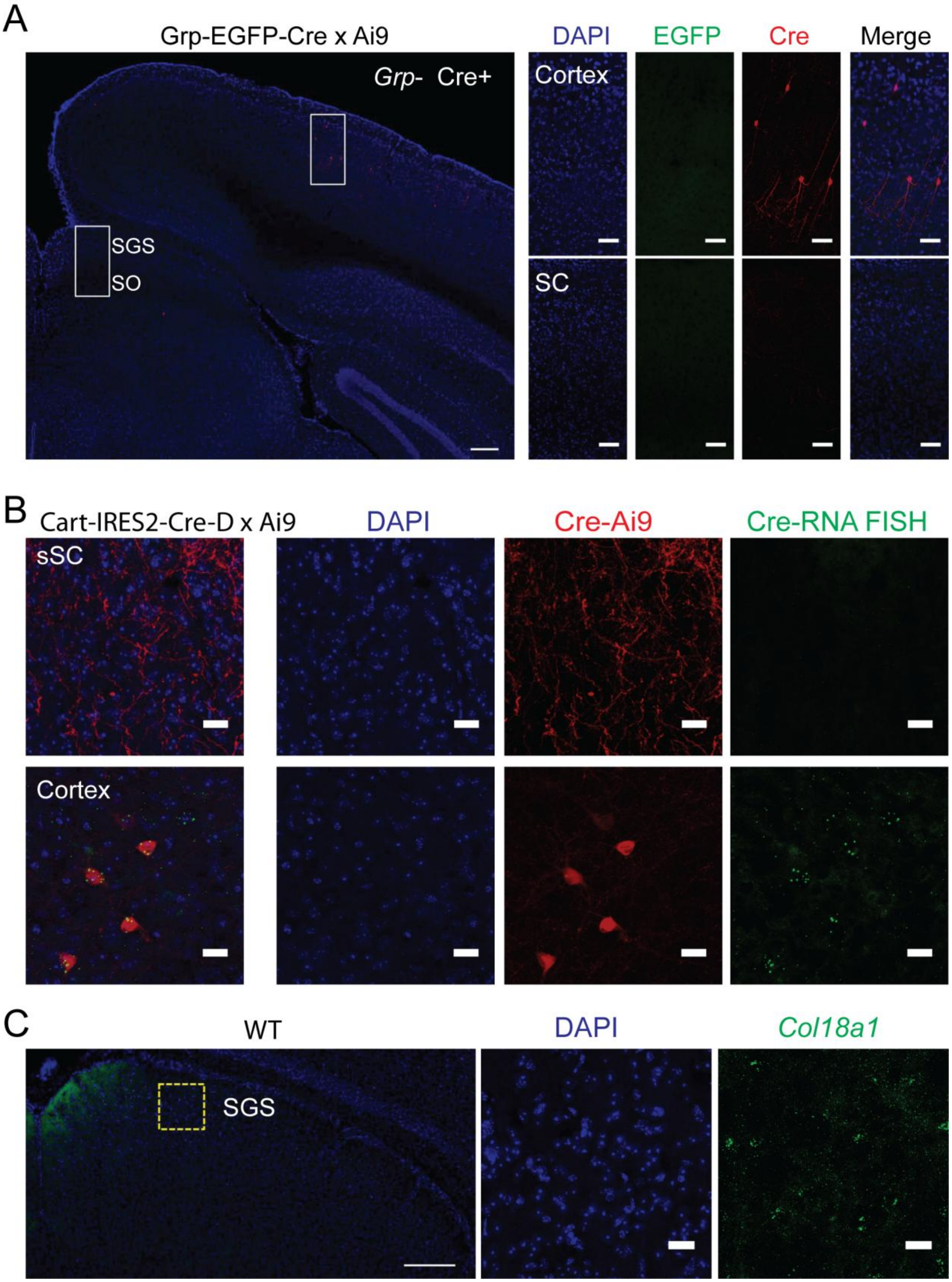
A
Grp-EGFP-Cre x Ai9
Grp- Cre+
SGS
SO
DAPI
EGFP
Cre
Merge
Cortex
SC
B
Cart-IRES2-Cre-D x Ai9
DAPI
Cre-Ai9
Cre-RNA FISH
sSC
Cortex
C
WT
SGS
DAPI
Col18a1

Figure S2. Characterization of mouse lines for other excitatory neuron types in the SC, Related to Figure 3-4

(A) Cre expression in the SC and cortex of Grp-EGFP-Cre x Ai9 mice (*Grp*-, *Cre*+ for genotyping). The area of white rectangle on the left is shown at a higher magnification with individual and merged channels to the right. Scale bars: 200 µm (left) and 50 µm (right).

(B) RNA FISH of *Cre* expression in the sSC and cortex of Cart-IRES2-Cre-D x Ai9 mice with merged channel on the left and individual channels on the right. Scale bars: 20 µm.

(C) RNA FISH of *Col18a1* in the SC of WT mice. The area of yellow square on the left is shown at a higher magnification with individual channels to the right. Scale bars: 200 µm (left) and 20 µm (right).

Table S1. Oligonucleotides used in this study, related to the STAR Methods

Oligonucleotides		
Mm-Slc17a6-C2	Advanced Cell Diagnostics	Cat #: 319171-C2
Mm-Slc17a6-T2	Advanced Cell Diagnostics	Cat #: 319171-T2
Mm-Npnt-C2	Advanced Cell Diagnostics	Cat #: 316771-C2
Mm-Tcf7l2-C3	Advanced Cell Diagnostics	Cat #: 466901-C3
Mm-Grp-C2	Advanced Cell Diagnostics	Cat #: 317861-C2
Mm-Cdh7-T3	Advanced Cell Diagnostics	Cat #: 520761-T3
Mm-Cartpt-C1	Advanced Cell Diagnostics	Cat #: 432001
Mm-Slc32a1-C1	Advanced Cell Diagnostics	Cat #: 319191
Mm-Cbln4-C3	Advanced Cell Diagnostics	Cat #: 428471-C3
Mm-Cbln4-T3	Advanced Cell Diagnostics	Cat #: 428471-T3
Mm-Col18a1-T9	Advanced Cell Diagnostics	Cat #: 483801-T9
